# A Temporal Credential-Based Mutual Authentication with Multiple-Password Scheme for Wireless Sensor Networks

**DOI:** 10.1371/journal.pone.0170657

**Published:** 2017-01-30

**Authors:** Xin Liu, Ruisheng Zhang, Qidong Liu

**Affiliations:** School of information Science & Engineering, Lanzhou University, Lanzhou, China; Nankai University, CHINA

## Abstract

Wireless sensor networks (WSNs), which consist of a large number of sensor nodes, have become among the most important technologies in numerous fields, such as environmental monitoring, military surveillance, control systems in nuclear reactors, vehicle safety systems, and medical monitoring. The most serious drawback for the widespread application of WSNs is the lack of security. Given the resource limitation of WSNs, traditional security schemes are unsuitable. Approaches toward withstanding related attacks with small overhead have thus recently been studied by many researchers. Numerous studies have focused on the authentication scheme for WSNs, but most of these works cannot achieve the security performance and overhead perfectly. Nam et al. proposed a two-factor authentication scheme with lightweight sensor computation for WSNs. In this paper, we review this scheme, emphasize its drawbacks, and propose a temporal credential-based mutual authentication with a multiple-password scheme for WSNs. Our scheme uses multiple passwords to achieve three-factor security performance and generate a session key between user and sensor nodes. The security analysis phase shows that our scheme can withstand related attacks, including a lost password threat, and the comparison phase shows that our scheme involves a relatively small overhead. In the comparison of the overhead phase, the result indicates that more than 95% of the overhead is composed of communication and not computation overhead. Therefore, the result motivates us to pay further attention to communication overhead than computation overhead in future research.

## Introduction

With the development of microelectronic, computer, and wireless communication techniques, multifunctional sensor nodes with small consumption have rapidly developed [1]. As a result, the Internet of Things has become increasingly popular. Wireless sensor networks (WSNs), which consist of a large number of sensor nodes (SNs), are widely used in various application fields, such as, environmental monitoring, military surveillance, nuclear-reactor control systems, vehicle safety systems, and medical monitoring [2, 3]. Although WSNs perform important functions in numerous application fields, the drawbacks of the network are evident. First, WSNs are often deployed in unattended environments [4] or enemy-controlled environments. Therefore, the networks are easily manipulated. Second, given their characteristics, WSNs consist of numerous resource-constrained nodes. The main limitation points are as follows [5]:

1. Given the low data-transfer rate, the short communication distance, and the harsh environment deployment, the transmission of WSNs is unreliable and has a higher energy costs.
2. Owing to the small size of SNs, each node is supplied with a small battery. WSNs are, however, always deployed in unattended environments or enemy environments; therefore, energy supplementation is impracticable.
3. As SNs use embedded processor and memory, only base computation capacity is available for processing. Therefore, the technology is limited by low computation and storage capacity.

The security of WSNs is related to sensitive data and safety of patients, and it can even escalate to national security. Compared with traditional networks, however, WSNs are vulnerable to various related attacks. Unfortunately, the information transmitted in WSNs is highly important and sensitive, so adversaries A can destroy WSNs or obtain confidential information from such networks. Therefore, the challenge and priority is to secure the performance of WSNs with small overhead, and this topic has recently been studied by many researchers. Authentication schemes have become the most important concern in the security of WSNs. In the last five years, numerous mutual-authentication and key agreement schemes have been published by researchers around the world and are discussed in the following subsection.

## Related Work

The authentication scheme for WSNs has recently been studied by many professors, and several investigations have surveyed the security of WSNs [3, 6–13]. These studies have analyzed the main problems faced by WSN security research and classified authentication schemes into two types: scheme-based asymmetric encryption and scheme-based symmetric encryption. The majority of the schemes aim to achieve improved security performance with small overhead. Nam et al. [14] proposed an anonymous scheme with lightweight computation. The group used elliptic curve cryptography for better security and focused on user anonymity. Watro et al. [15] proposed a security scheme of mutual authentication with RSA cryptosystem and Diffie—Hellman key agreement. Wong et al. [16] proposed another password-based authentication scheme that only uses hash functions. The scheme proposed by Wong et al. is therefore more efficient than Watro et al.’s schemes. However, their scheme is vulnerable to numerous attacks, as proven by M. L. Das et al. [17], who proposed a two-factor scheme with a password and a smart card (SC). Although vulnerable to numerous attacks, the scheme prompted other researchers to improve the two-factor authentication for WSNs. Xue et al. [18] proposed temporal credential authentication for WSNs. This scheme allows the gateway nodes (GW) to issue a temporal credential to users and SNs for mutual authentication. The scheme is efficient because it only uses the hash function and XOR operation. Jiang et al. [19] concluded that Xue et al.’s scheme cannot withstand the privileged insider, weak stolen smart card, identity guessing, and tracking attacks. Then, Jiang et al. proposed a two-factor user authentication scheme with unlinkability for WSNs. Despite presenting an improvement on the weakness of Xue et al.’s approach, Jiang et al.’s scheme is also vulnerable to privileged insider attacks and presents several drawbacks, as proven by A. K. Das [20]. The scheme proposed by A. K. Das used biometrics as the third factor for user authentication and improved the weakness of the scheme by Xue et al. He et al. [21] also found drawbacks in Xue et al.’s scheme. Through their analysis, the team found that Xue et al.’s scheme is vulnerable to offline password guessing, user impersonation, and modification attacks. Thereafter, He et al. proposed a temporal credential authentication with pseudo identity for WSNs. The scheme proposed by Khan and Alghathbar [22] indicated that M. L. Das’s scheme cannot withstand bypassing attacks and is vulnerable to privileged insider attacks. Sun et al. [23] concluded that Khan and Alghathbar’s scheme is vulnerable to GW impersonation and other related attacks. Sun et al. proposed a scheme to improve the weakness of Khan and Alghathbar’s scheme and determined that their scheme had low overhead cost.

Key establishment is the central problem in authentication schemes [24]. Diffie and Hellman proposed the revolutionary introduction of the key establishment protocol [25] and Bellare and Rogaway proposed a model of authentication and key distribution that is widely accepted [26–28]. Choo et al. discovered that all secure key distribution protocols should use partnering definitions based on session identifiers [29] and that session identifiers should also be included within the protocol specification [30]; the secure protocols should construct the session keys using the identities of participants, unique session identifiers and ephemeral-long-term shared secrets [31]; and any entity authentication and key establishment protocol should provide rigorous proof of security based on their meticulous research [32]. They also carefully researched the subtle differences between the well-known models and contributed a better understanding of proof models for key establishment protocols [33]. Based on the careful study, Choo and Hitchcock proposed that the proof models allow different options for the key-sharing requirement in formulation [34]. Numerous researchers have worked on fulfilling this requirement, so listing these works in our paper is unnecessary.

## Our Contribution

In this paper, we propose a temporal credential-based mutual authentication with a multiple-password scheme for WSNs. Comparison with other related works shows that our proposed scheme exhibits improved security performance with low overhead. The major contributions are described as follows.

1. We perform user authentication without any GW consumption which presents better efficiency and security performance, as proven by A. K. Das and Amin et al.’ s research [20, 35], they bind U with ID_SC_ so that the scheme can reliably withstand D-DOS attacks that are launched by inputting wrong passwords [20] as well as withstand same-login ID attacks [22, 36].
2. We use multiple passwords to authenticate the legality of the user identity. We select all user-inputting passwords, the sequence of passwords, and the number of passwords n as the factors to verify the identity of the user. This innovation not only presents the same security performance as the three-factor authentication based on biometrics but also exhibits a more efficient performance than biometric authentication. This approach overcomes several weaknesses of biometric authentication, which is unsuitable for WSNs. These disadvantages include high noise data rate, false non-match rate, false match rate, intraclass variations, non-universality, spoof attacks [37], high biometric error rate, stolen biometric features attacks [38], and high consumption [20, 39].
3. Through detailed comparison, we found that communication overhead accounts for the majority of the overhead. Most of the related studies, which were concerned only with computation overhead, are not comprehensive. Therefore, more attention should be paid to communication overhead than to computation overhead to evaluate the performance of any scheme in future research.

## Notations in This Paper

The notations used in this paper are described as follows.

GW: a gateway node
U: the user
SN: the sensor node
SC: the smart card of U
A: the adversary
ID_U_: the identity of U
ID_GW_: the identity of GW
ID_SC_: the identity of SC
ID_SN_: the identity of SN
PW_U_: the password of U
n: the number of passwords
k_i_, k_GW_, k_i_: the secret number for U,GW,SN respectively
e_i_, PK_GW_, PK_j_: the protected information for the secret number of U,GW,SN, respectively
V_i_: the verification information of U
DID_SC_, PID_j_: the pseudonym of SC,SN, respectively
T_U_, T_GW_, TS: the current timestamp
RPW_i_: the protected information for the multiple password
PTC_i_, PTC_j_: the protected temporal credential of U, SN, respectively
SK: the session key in the future
σ_U_, σ_GW_: the HMAC output with secret keys k_UG_, k_GS_, respectively
(Mac, Ver): a keyed-hashing for message authentication codes
(Enc, Dec): symmetric encryption/decryption functions
H(·): hash function
∥: bitwise concatenation operation

## Review of Nam et al.’s Scheme

In this section, we review Nam et al.’s scheme in detail. The scheme consists of three phases: the registration phase, the login phase, and the authentication and key exchange phase [14]. Nam et al.’s scheme stores an elliptical curve group *G* with generator *P* of prime order *q*; MAC function ∑ = (Mac, Ver) [40, 41]; symmetric encryption and decryption functions Δ = (Enc, Dec); and three hash functions, *H*, *J*, and *I* in each entity (we use only *H* to represent the hash function in this paper). After finishing these tasks, GW selects two random numbers, $y\in Z_{q}^{*}$ and, z ∈ {0, 1}^k^, computes Y = yP with k_GS_ = h(ID_SN_ ∥ z) as the public key and shares a secret key with SN.

### Registration phase

A user U registers his identity ID_U_ and password PW_U_ through the following steps.

1. A user U registers the identity ID_U_ and password PW_U_ and submits ID_U_ to the GW.
2. GW computes EID_U_ = Enc_z_(ID_U_ ∥ ID_GW_) with the key z and sends {EID_U_, Y, ID_GW_, G, P, ∑, Δ, H} to U. U stores these messages in the SC.
3. U computes XEID_U_ = EID_U_ ⊕ h(ID_U_ ∥ PW_U_) to replace EID_U_.

### Login, authentication, and key exchange phase

In these phases, U, GW, and SN authenticate each other through the following, and the session key SK is generated. The details of these phases are described as follows:

1. U inserts his SC and inputs the identity ID_U_ and password PW_U_. Then, SC retrieves the current timestamp T_U_ and gets two random numbers $x\in Z_{q}^{*}$, k_US_ ∈ {0, 1}^k^. SC performs a series of calculations as follows. K_UG_ = xY, X = xP, k_UG_ = h(T_U_ ∥ X ∥ Y ∥ K_UG_), EID_U_ = XEID_U_ ⊕ h(ID_U_ ∥ PW_U_), $C_{U}\;=\;{Enc}_{k_{UG}}({ID}_{U}∥{EID}_{U}∥k_{US})$ and $\sigma_{\text{U}}={\text{Mac}}_{{\text{k}}_{\text{UG}}}({\text{ID}}_{\text{GW}}∥{\text{ID}}_{\text{SN}}∥{\text{T}}_{\text{U}}∥{\text{C}}_{\text{U}})$. Finally, U sends (T_U_, ID_SN_, X, C_U_, σ_U_) to GW.
2. Upon receiving these message, GW checks the freshness of T_U_, if T_U_ is not fresh, GW discards the session. Otherwise, GW checks whether ${Ver}_{k_{UG}}({ID}_{GW}∥{ID}_{SN}∥T_{U}∥C_{U},\sigma_{U})\;$ is equal to 1, where k_UG_ = h(T_U_ ∥ X ∥ Y ∥ K_UG_) and K_UG_ = yX. If it is not equal, GW discards the session. Otherwise, GW uses the key k_UG_ to decrypt C_U_ to get ID_U_ and EID_U_. GW uses the key z to decrypt EID_U_ to get ${ID}_{U}^{'}$. Then, GW checks whether ID_U_ is equal to ${ID}_{U}^{'}$. If they are equal, GW computes $C_{GW}\;=\;{Enc}_{k_{GS}}(k_{US})$ and $\sigma_{\text{GW}}={\text{Mac}}_{{\text{k}}_{\text{GS}}}({\text{ID}}_{\text{GW}}∥{\text{ID}}_{\text{SN}}∥{\text{T}}_{\text{GW}}∥{\text{T}}_{\text{U}}∥{\text{C}}_{\text{GW}})$, where T_GW_ is the current TS. Finally, GW sends (ID_GW_, T_GW_, T_U_, C_GW_, σ_GW_) to SN.
3. Upon receiving these messages, SN first checks the freshness of T_GW_. If T_GW_ is not fresh, SN aborts the session. Otherwise, SN checks whether ${Ver}_{k_{GS}}({ID}_{GW}∥{ID}_{SN}∥T_{GW}∥T_{U}∥C_{GW},\sigma_{GW})$ is equal to 1. If it is not equal, SN aborts the session. Otherwise, SN decrypts C_GW_ with the key k_GS_ to get k_US_. Then, SN computes SK = h(k_US_ ∥ T_U_ ∥ ID_SN_) and ρ_SN_ = h(k_US_ ∥ T_U_ ∥ ID_SN_). Finally, SN sends ρ_SN_ to the user U.
4. Upon receiving messages, the user checks whether ρ_SN_ is equal to h(k_US_ ∥ ID_SN_ ∥ T_U_). If they are not equal, U aborts the session. Otherwise, U computes SK = h(k_US_ ∥ T_U_ ∥ ID_SN_) as the SK.

### Password update phase

In this phase, Nam et al. have designed an interactive password update phase as follows:

1. U inserts his SC and inputs ID_U_, PW_U_, and new password ${PW}_{U}^{'}$.
2. SC completes a series of calculations with the random $x\in Z_{q}^{*}$ and timestamp T_U_ as follows. k_UG_ = xY, X = xP, k_UG_ = h(T_U_ ∥ X ∥ Y ∥ K_UG_), EID_U_ = XEID_U_ ⊕ h(ID_U_ ∥ PW_U_) $C_{U}\;=\;{Enc}_{k_{UG}}({ID}_{U}∥{EID}_{U})$. Then, SC sends (T_U_, C_U_, X) to the GW.
3. Upon receiving these messages, GW rejects the request if T_U_ is not fresh. Otherwise, GW computes k_UG_ = h(T_U_ ∥ X ∥ Y ∥ K_UG_) and K_UG_ = yX. Then, GW uses the key k_UG_ to decrypt C_U_ to get ID_U_ and EID_U_. GW decrypts EID_U_ with the key z to get another ${ID}_{U}^{'}$. GW checks whether ID_U_ is equal to ${ID}_{U}^{'}$. If they are equal, GW computes ρ_GW_ = h(k_UG_ ∥ X ∥ ID_U_ ∥ ID_GW_) and sends ρ_GW_ to SC.
4. SC checks whether ρ_GW_ is equal to h(k_UG_ ∥ X ∥ ID_U_ ∥ ID_GW_). If they are not equal, SC aborts the session. Otherwise, SC computes ${XEID}_{U}\;=\;{EID}_{U}⊕h({ID}_{U}∥{PW}_{U}^{'})$ and finishes the password update phase.

## Security Analysis of Nam et al.’s Scheme

In this section, we comprehensively analyze the security performance of Nam et al.’s scheme. During the analysis, several weaknesses of the scheme were identified. Nam et al.’s scheme ensures user anonymity and uses the elliptical curve computational Diffie—Hellman (ECCDH) protocol and authenticated key exchange (AKE) to fulfill the security function. However, further analysis shows that the scheme is vulnerable to the following threats.

### D-DOS attacks

In the authentication and key exchange phase or password update phase of Nam et al.’s scheme, SC and GW need to execute numerous complex computations to verify the identity of U. To fulfill this task, SC and GW have to execute the hash function three times, encryption once, decryption twice, and MAC calculation and Ver calculation twice. Following several studies [3, 20, 42], we assume that an adversary A would start a D-DOS attack that is launched by persistently inputting a wrong ID_U_ or wrong PW_U_. According to Nam et al. [14] and the reference basis that is analyzed in this paper, each verification needs approximately 9.5 hash calculations, wasting 0.00304 s and costing 0.073 mJ of WSNs. A would not be suspended until the energy of GW is depleted [42].

Based on the preceding discussion, Nam et al.’s scheme is vulnerable to D-DOS attacks, and adversary A can easily drain the batteries in the login phase.

### Online guessing attacks

In the authentication and key exchange phase, we assume that A eavesdrops on the communication channel [43]. A can obtain the secret key *k*_US_ and compute the SK with an online guessing attack through the following steps:

1. A obtains *T*_U_, ID_SN_, and *ρ*_SN_ by intercepting channels U → GW, GW → SN, and SN → U.
2. A guesses the *k*_US_ from the directory.
3. A verifies whether *h*(k_US_ ∥ ID_SN_ ∥ T_U_) is equal to *ρ*_SN_. If both numbers are the same, A obtains k_US_. Otherwise, A repeats steps 2 and 3 until the correct k_US_ is guessed.
4. After obtaining *k*_US_, A computes SK = h(k_US_ ∥ T_U_ ∥ ID_SN_) to obtain the SK.

According to the preceding discussion, we conclude that A can obtain the secret key k_US_ and compute the SK by online guessing attacks. These findings prove that Nam et al.’s scheme is vulnerable to online guessing attacks.

### Lost password threat

Numerous approaches, such as the hit library attack and social engineering [44, 45], can be used to obtain user passwords. The lost password threat is currently popular and is a deadly threat to any one-password-based authentication, including WSNs. If the adversary A obtains the commonly used passwords of U by other methods, we can see that the authentication scheme encounters a considerable threat.

### Replay attacks

In the authentication and key exchange phase, we assume that an adversary A intercepts the message *ρ*_SN_. Then, A sends *ρ*_SN_ to *U*. As *U* does not check the freshness of T, U cannot realize that A has already obtained the *ρ*_SN_, therefore proving that Nam et al.’s scheme is vulnerable to replay attacks.

### Impersonation attacks

In the authentication and key exchange phase, SN authentication verifies whether the identity of GW is invalid. Furthermore, U does not authenticate the validity of SN. A can start the impersonation attack by forging GW and SN as in the following steps:

1. A intercepts ID_GW_, *T*_GW_, *T*_U_, *C*_GW_, and σ_GW_ from the communication channel GW → SN.
2. A sends ID_GW_, *T*_GW_, *T*_U_, *C*_GW_, and *σ*_GW_ to SN.
3. A passes the MAC, and A is believed to be the real GW.

According to the preceding discussion, as *U* does not check the freshness of T, we can safely conclude that Nam et al.’s scheme is vulnerable to impersonation attacks. The detailed security analysis is described in Table 1.

**Table 1:** The security comparison with other schemes.

	SSCA	NCA	PIA	MA	UA	ONGA	OFPGA	RA	MITMA	LPT	DDA	MSNA	TFS	IOM	IGA	SKA	PUP	DNAP
D.B.He	yes	no	no	yes	yes	no	yes	yes	yes	no	no	n/a	no	yes	yes	yes	no	no
A.K.Das	yes	no	yes	yes	yes	yes	yes	yes	yes	no	yes	no	yes	no	yes	yes	yes	yes
J.H.Nam	no	yes	yes	no	yes	no	no	no	no	no	no	n/a	no	yes	no	yes	yes	no
K.XUE	no	no	no	yes	no	yes	no	yes	yes	no	no	n/a	no	no	no	yes	yes	no
Q.Jiang	no	no	no	no	yes	no	yes	yes	yes	no	no	n/a	no	yes	no	yes	no	no
M.L.Das	no	no	no	yes	no	no	yes	yes	yes	no	no	n/a	no	no	no	no	no	no
Ours	yes	yes	yes	yes	yes	yes	yes	yes	yes	yes	yes	yes	yes	yes	yes	yes	yes	yes

SSCA: Stolen smart card attack; NCA: Nodes captured attack; PIA: Privileged insider attack; MA: mutual authentication; UA: Anonymity; ONGA: Online guessing attack OFPGA: Off-line password guessing attack; RA: Replay attack; MITMA: Man-in-the-middle attack; LPT: Lost password threat; DDA: D-Dos attack; MSNA: Malicious sensor node attacks; TFS: Three-factor security; IOM: Integrity of message; IGA: identity guessing attack; SKA: session key agreement; SKA: session key agreement; PUP: password updated phase; DNAP: dynamic node addition phase

## Our Proposed Scheme

In this section, we propose a temporal credential-based mutual authentication with multiple-password scheme for WSNs. The temporary SK has many advantages relative to using long-term keys according to Choo’s research [46]. Our scheme not only inherits the excellent properties of Nam et al.’s scheme but also improves upon the weaknesses of their scheme. As our scheme uses multiple passwords to replace Tate-pairing computation and the fuzzy extractor function, our scheme can achieve the same security performance with smaller overhead [47].

Unlike Nam et al.’s scheme, our proposed scheme consists of five phases: registration phase, login phase, authentication and key exchange phase, password update phase, and dynamic-node addition phase. These phases are described in detail as follows.

### Registration phase

In this phase, we register a legal user, U, and sensor nodes, SN. This concept has already been presented in other studies [18, 21]. The registration phase is executed in a rigorously secure environment prior to the deployment of WSNs. Before registration, GW assigns the unique identities, namely, ID_SN_, ID_SC_, and ID_GW_, to SNs, SC, and the GW respectively. Then, GW randomly generates a secret number, *k*_GW_. Finally, the hash function-H(∙); message authentication check scheme MAC(∙); and Ver(∙) are stored in SC, GW, and SN. The registration phase is described in detail as follows.

#### Registration phase for legal user

In this phase, we register the legal user U through the following steps.

1. U inserts his SC and inputs his multiple-password PW_1_, PW_2_ ⋯PW_n_. U generates a random secret number K_i_ and gets the unique identifier ID_SC_. U computes RPW_i_ = H(ID_SC_ ∥ PW_1_ ∥ PW_2_ ∥ ⋯∥PW_n_ ∥ n ∥ k_i_) and retrieves the timestamp TS_1_. Finally, U sends (RPW_i_, TS_1_, ID_SC_) to GW.
2. Upon receiving the message, GW checks the freshness of TS_1_. If TS_1_ is not fresh, GW rejects the request. Otherwise, GW gets the unique identifier ID_GW_. Then, GW computes TC_i_ = H(k_GW_ ∥ ID_GW_ ∥ ID_SC_), PTC_i_ = TC_i_ ⊕RPW_i_, and PK_GW_ = PTC_i_⊕k_GW_. Then, GW retrieves the current timestamp TS_2_. Finally, GW stores the tuple (ID_GW_, ID_SC_, PK_GW_) in the verification table and sends (PTC_i_, TS_2_, ID_GW_) to U.
3. Upon receiving the message, U checks the freshness of TS_2_. If TS_2_ is not fresh, U rejects the request. Otherwise, U computes e_i_ = k_i_⨁H(n ∥ PW_1_ ∥ PW_2_ ∥⋯∥ PW_n_), V_i_ = H(e_i_ ∥ RPW_i_ ∥ ID_SC_ ∥ k_i_ ∥ n). Finally, U stores (e_i_, V_i_, PTC_i_, ID_SC_, ID_GW_) in the SC.

**Fig 1:**
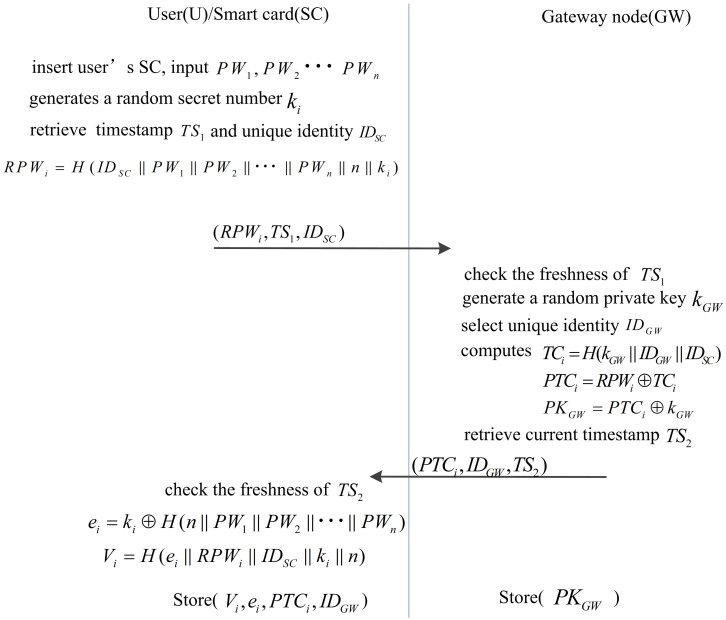
The registration phase for user of our scheme.

In this phase, adversary A cannot restore the sensitive number because of the property of the hash function [48–50] and the confidentiality property of the XOR operation [51–53], as well as the information stored in GW and SC. The random secret numbers *k*_i_ and *k*_GW_ are not stored in GW. This phase is shown in Fig 1.

#### Registration for sensor node

In our scheme, each legal SN is required to register in GW so that we can verify the legal SN and add the new SN to WSNs in the future. Before SN registration, the legality of U should be verified. The steps are as follows.

1. SN generates a random secret number k_j_ and gets the unique identifier ID_SN_. Then, SN computes PID_j_ = H(ID_SN_ ∥ k_j_), PK_j_ = PID_j_⊕k_j_ and replaces ID_SN_ with PID_j_. Finally, SN retrieves timestamp TS_3_ and sends (PID_j_, TS_3_) to GW.
2. Upon receiving the message, GW checks the freshness of TS_3_. If TS_3_ is not fresh, GW rejects the request. Otherwise, GW computes TC_j_ = H(k_GW_ ∥ PID_j_), PTC_j_ = TC_j_⊕PID_j_. Then, GW retrieves the timestamp TS_4_ and stores PID_j_. Finally, GW sends (TS_4_, PTC_j_) to SN.
3. Upon receiving the message, SN checks the freshness of TS_4_. If TS_4_ is not fresh, GW rejects the request. Otherwise, SN stores (PK_j_, PTC_j_).

**Fig 2:**
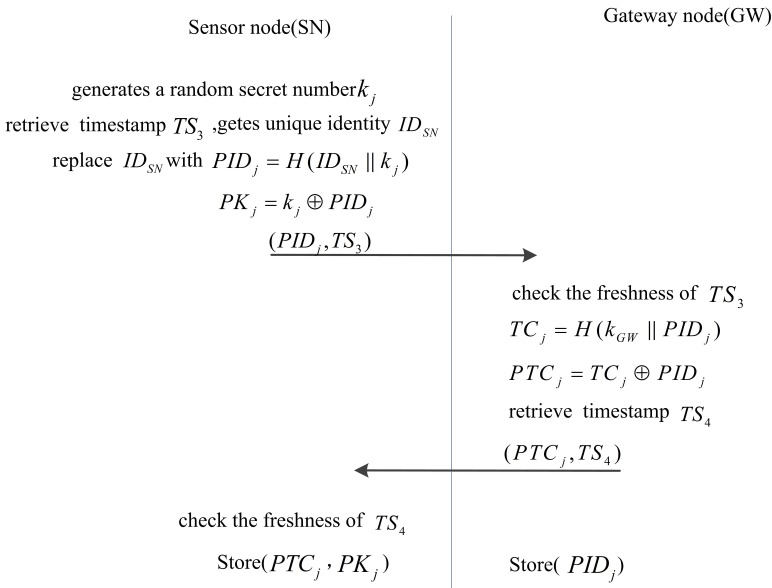
The registration phase of sensor node of our scheme.

In this phase, different SNs possess different PID_j_ and PK_j_, and the random secret number K_j_ is not stored in SN. Therefore, our scheme can withstand node capture attacks, as analyzed in the security analysis section. This phase is shown in Fig 2. After finishing the entire registration scheme, GW deletes k_GW_, SC deletes K_i_, and SN deletes K_j_ before the deployment of WSNs.

### Login phase

**Fig 3:**
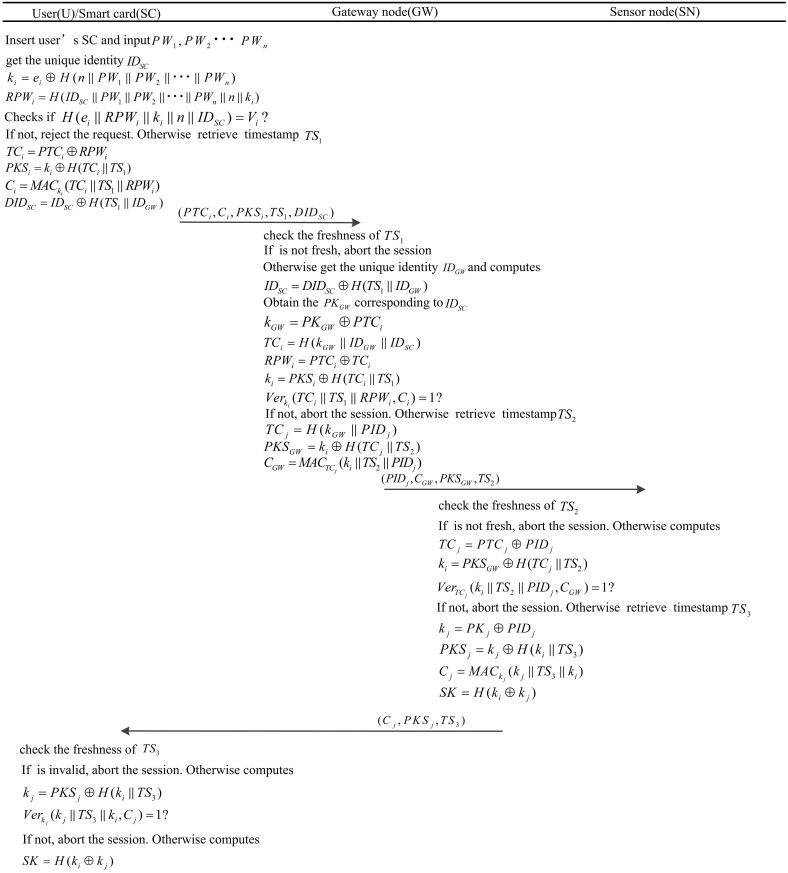
The login, authentication and key exchange phase of our scheme.

The login phase procedure is described in detail as follows. If U attempts to login to WSNs and obtains data from SN, the following steps are executed. This phase is shown in Fig 3.

1. U inserts his SC and inputs the registered multiple-password PW_1_, PW_2_ ⋯PW_n_.
2. SC gets the unique identifier ID_SC_ and computes k_i_ = e_i_⨁H(n ∥ PW_1_ ∥ PW_2_ ∥⋯∥ PW_n_), RPW_i_ = H(ID_SC_ ∥ PW_1_ ∥ PW_2_ ∥ ⋯∥PW_n_ ∥ n ∥ k_i_).
3. SC checks whether H(e_i_ ∥ RPW_i_ ∥ k_i_ ∥ n ∥ ID_SC_) is equal to V_i_. If it is not equal, SC rejects the request. Otherwise, SC retrieves timestamp TS_1_ and computes TC_i_ = PTC_i_⊕RPW_i_, PKS_i_ = k_i_⊕H(TC_i_ ∥ TS_1_), $C_{i}\;=\;{MAC}_{k_{i}}({TC}_{i}∥{TS}_{1}∥{RPW}_{i})$, DID_SC_ = ID_SC_⊕H(TS_1_ ∥ ID_GW_).
4. Finally, U sends (PTC_i_, C_j_, PKS_i_, TS_1_, DID_SC_) to GW.

### Authentication and key exchange phase

In this phase, we describe the authentication mechanism through U, GW, and SC. The mechanism achieves mutual authentication and generates the SK, for future use. The details are presented as follows.

1. Upon receiving the message, GW checks the freshness of TS_1_. If it is not fresh, GW aborts the session. Otherwise, GW retrieves the unique identity ID_GW_ and computes ID_SC_ = DID_SC_⨁H(TS_1_ ∥ ID_GW_), GW obtains the PK_GW_ corresponding to ID_SC_ in the verification table. Then, GW computes k_GW_ = PK_GW_⨁PTC_i_, TC_i_ = H(k_GW_ ∥ID_GW_), RPW_i_ = PTC_i_⨁TC_i_, and k_i_ = PKS_i_⨁ (TC_i_ ∥ TS_1_). GW checks whether Ver_ki_(TC_i_ ∥ TS_1_ ∥ RPW_i_, C_i_) is equal to 1. If it is not equal, GW aborts the session. Otherwise, GW retrieves timestamp TS_2_ and computes TC_j_ = H(k_GW_ ∥PID_j_), PKS_GW_ = k_i_⨁H(TC_j_ ∥ TS_2_),$C_{GW}\;=\;{MAC}_{{TC}_{j}}(k_{i}∥{TS}_{2}\;∥{PID}_{j})$. Finally, GW sends (PID_j_, C_GW_, PKS_GW_, TS_2_) to SN.
2. Upon receiving the message, SN checks the freshness of TS_2_. If it is not fresh, SN aborts the session. Otherwise, SN computes TC_j_ = PTC_j_⨁PID_j_, k_i_ = PKS_GW_⨁H(TC_j_ ∥TS_2_). Then, SN checks whether ${Ver}_{{TC}_{j}}(k_{i}∥{TS}_{2}∥{PID}_{j},C_{GW})$ is equal to 1. If it is not equal, SN aborts the session. Otherwise, SN retrieves timestamp TS_3_ and computes k_i_ = PK_i_⨁PID_j_, PKS_j_ = k_j_⨁H(k_i_ ∥TS_3_),$C_{j}\;=\;{MAC}_{k_{j}}(k_{j}∥{TS}_{3}∥k_{i})$ and SK = H(k_i_⨁k_j_) as the SK. Finally, SN sends (C_j_, PKS_j_, TS_3_) to U.
3. Upon receiving the message, U checks the freshness of TS_3_. If it is not fresh, U aborts the session. Otherwise, the SC of U computes k_j_ = PKS_j_⨁H(k_i_ ∥TS_3_). Then SC checks whether ${Ver}_{k_{j}}(k_{j}∥{TS}_{3}∥k_{i},C_{j})$ is equal to 1? If it is not equal, SC aborts the session. Otherwise, SC computes SK = H(k_i_⨁k_j_) as the SK for the future.

In this phase, our proposed scheme not only achieves mutual authentication and key establishment but also checks the integrity of the message. Each message authentication check function in U, SN, and GW uses different secret encryption keys for secure communication [3]. The detailed security performance of our scheme is discussed in the security analysis section, and the authentication and key exchange phase is shown in Fig 3.

### Password updated phase

For security reasons, U needs to change his/her password periodically. In this phase, we propose the password-updating phase to change the password of U and U can change the sequence of passwords and the number of passwords as the new identity characteristics with minimal consumption. The details of this phase are described as follows.

1. U inserts his SC and inputs the older multiple-password PW_1_, PW_2_ ⋯PW_n_.
2. SC gets the unique identifier ID_SC_ and computes k_i_ = e_i_⨁H(n ∥ PW_1_ ∥ PW_2_ ∥⋯∥ PW_n_), RPW_i_ = H(ID_SC_ ∥ PW_1_ ∥ PW_2_ ∥ ⋯∥PW_n_ ∥ n ∥ k_i_).
3. SC checks whether H(e_i_ ∥ RPW_i_ ∥ k_i_ ∥ n ∥ID_SC_) is equal to V_i_. If it is not equal, SC rejects the request. Otherwise, SC computes TC_i_ = PTC_i_⨁RPW_i_. Then, U inputs his new multiple-password ${PW}_{1}^{new},{PW}_{2}^{new}\cdots {PW}_{m}^{new}$.
4. After inputting the new multiple-password, SC computes ${RPW}_{i}^{new}\;=\;H({ID}_{SC}∥{PW}_{1}^{new}∥{PW}_{2}^{new}∥\cdots ∥{PW}_{m}^{new}∥m∥k_{i})$, ${PTC}_{i}^{new}\;=\;{TC}_{i}⨁{RPW}_{i}^{new},\;{\;e}_{i}^{new}\;=\;k_{i}⨁H(m∥{PW}_{1}^{new}∥{PW}_{2}^{new}∥\cdots ∥{PW}_{m}^{new})$, $V_{i}^{new}\;=\;H({\;e}_{i}^{new}∥{RPW}_{i}^{new}∥{ID}_{SC}∥k_{i}∥m)$. U sends PTC_i_, ${PTC}_{i}^{new}$, and current TS to GW. Finally, SC replaces (e_i_, V_i_, PTC_i_) with(${\;e}_{i}^{new},V_{i}^{new},\;{PTC}_{i}^{new}$).
5. Upon receiving ${PTC}_{i}^{new}$, GW checks the freshness of TS. If it is not fresh, GW rejects the request. Otherwise, GW computes k_GW_ = PK_GW_⨁PTC_i_, ${PK}_{GW}^{new}\;=\;{PTC}_{i}^{new}⨁k_{GW}$. Then, GW replaces PK_GW_ with ${PK}_{GW}^{new}$.

### Dynamic node addition phase

New node deployment is inevitable in WSNs because nodes may be lost, exhausted, or destroyed [54]. In this phase, our proposed scheme allows U to add new SN to WSNs after deployment. Our scheme strictly requires that the dynamic node addition phase must be executed by the legal user. Thus, our scheme must initially verify the legality of U. We assume that a new sensor node SN^new^ is going to join the WSNs, and the following steps must be executed.

1. U inserts his SC and inputs the registered multiple-password PW_1_, PW_2_ ⋯PW_n_.
2. SC gets the unique identifier ID_SC_ and computes k_i_ = e_i_⊕H(n ∥ PW_1_ ∥ PW_2_ ∥⋯∥ PW_n_) and RPW_i_ = H(ID_SC_ ∥ PW_1_ ∥ PW_2_ ∥ ⋯∥PW_n_ ∥ n ∥ k_i_).
3. SC checks whether H(e_i_ ∥ RPW_i_ ∥ k_i_ ∥ n ∥ID_SC_) is equal to V_i_. If it is not equal, SC rejects the request. Otherwise, SC sends PTC_i_ and the current TS to GW.
4. GW checks the freshness of TS. If it is not fresh, GW rejects the request. Otherwise, GW computes k_GW_ = PK_GW_⨁PTC_i_ and assigns the new unique identifier ${ID}_{SC}^{new}$ to SN^new^ via a secure channel.
5. Finally, SN^new^ executes the registration phase for the sensor node.

Note that in this phase, the dynamic addition phase must be executed by a legal U that is authenticated by SC. This mechanism is able to withstand malicious sensor node attacks.

## Security Analysis

In this section, we analyze the security performance of our proposed scheme by both formal and informal analyses. We assume that A threatens the security of WSNs. Based on the existing defined models of adversary capabilities that are widely accepted [26, 27, 55, 56], and we conclude that A possesses the following hacking capabilities: (1) intercept the transmitted message via the channel [3, 6]; (2) use power analysis attacks to obtain the information stored in SC [57, 58] and use sensor node capture attack to obtain the information stored in SN [59–61]; (3)use dictionary attacks to guess numbers [43]; (4) posses the right to access the gateway station because he/she is a privileged user [40]; and (5) obtain the used passwords of U through other methods. We assume that sensitive information (PW_1_, PW_2_ ⋯PW_n_, n, k_i_, k_j_, k_GW_, TC_j_, TC_i_, SK) is attractive to A. Our goal is to prevent the sensitive information from being extracted by A. Thus we carefully analyzed the security performance of our proposed scheme using BAN-logic [62], which is popularly used to ensure the security of communication and session key agreement. The details of our analysis are described as follows.

### Formal analysis based on BAN-logic

In this section, we use BAN-logic to analyze the security of our proposed scheme. The notations of BAN-logic are defined as follows, where P denotes the principal as well as, X and Y denote the statements.

P |≡X: P believes X
P ⊲ X: P sees X
P | ∼ X: P once said X
P ⇒ X: P has jurisdiction over X
#(X): X is fresh
(X, Y): The formulae X or Y is one part of the formulae (X, Y)
< X >_Y_: X combined with Y
{X}_K_: X is encrypted under the key K
(X)_K_: X is hashed with the key K
$P\overset{K}{↔}\;Q$: P and Q communicate via shared key K
SK: The session key between U and SN
$P\overset{X}{\Leftrightarrow }Q$: The formulae X is known only to P and Q

Some main logical postulates of the BAN-logic are as follows:

The Message-meaning rule: $\frac{P\;|\equiv P\overset{K}{↔}\;Q,P\;⊲{\left\{X\right\}}_{K}}{P\;|\equiv Q|∼X}$,$\;\frac{P\;|\equiv P\overset{X}{\Leftrightarrow }Q,\;P\;⊲{<X>}_{Y}}{P\;|\equiv Q|∼X}$
The nonce-verification rule: $\frac{P\;|\equiv ⋕(X),P\;|\equiv Q|∼X}{P\;|\equiv Q|\equiv X}$
The jurisdiction rule: $\frac{P\;|\equiv Q\Rightarrow X,P\;|\equiv Q|\equiv X,}{P\;|\equiv X}$
The belief rule:$\;\frac{P\;|\equiv X,\;P\;|\equiv Y,}{P\;|\equiv (X,Y)}\;\frac{P\;|\equiv (X,Y)}{P\;|\equiv X},\;\frac{P\;|\equiv Q|\equiv (X,Y)}{P\;|\equiv Q|\equiv X}$
The freshness rule: $\frac{P\;|\equiv ⋕(X)}{P\;|\equiv ⋕(X,Y)}$
The session key rule: $\frac{P\;|\equiv ⋕(X),P\;|\equiv Q|\equiv X}{P\;|\equiv P\overset{K}{↔}\;Q}$

In order to prove the security of proposed scheme, the follow goals of BAN-logic must be satisfied.

Goal 1 $U\;|\equiv (U\overset{SK}{↔}\;SN)$
Goal 2 $U\;|\equiv SN|\equiv (U\overset{SK}{↔}\;SN)$
Goal 3 $SN|\equiv (U\overset{SK}{↔}\;SN)$
Goal 4 $SN|\equiv U|\equiv (U\overset{SK}{↔}\;SN)$
Goal 5 $U|\equiv (U\overset{k_{i}}{↔}\;SN)$
Goal 6 $U\;|\equiv SN|\equiv (U\overset{k_{i}}{↔}\;SN)$
Goal 7 $SN|\equiv (U\overset{k_{i}}{↔}\;SN)$
Goal 8 $SN\;|\equiv U|\equiv (U\overset{k_{i}}{↔}\;SN)$

**First**, the initial status of our scheme is made according to the following assumptions:

A_1_: U |≡⋕(TS_1_)
A_2_: U |≡⋕(TS_3_)
A_3_: $U\;|\equiv U\overset{{TC}_{i}}{↔}\;GW$
A_4_: $U\;|\equiv U\overset{{RPW}_{i}}{⟷}\;GW$
A_5_: $U\;|\equiv SN\Rightarrow U\overset{SK}{↔}\;SN$
A_6_: $U\;|\equiv SN\Rightarrow U\overset{k_{i}}{↔}\;SN$
A_7_: $U\;|\equiv SN\Rightarrow U\overset{k_{j}}{↔}\;SN$
A_8_: GW|≡⋕(TS_1_)
A_9_: GW|≡⋕(TS_2_)
A_10_: $GW\;|\equiv U\overset{{TC}_{i}}{↔}\;GW$
A_11_: $GW\;|\equiv U\overset{{RPW}_{i}}{⟷}\;GW$
A_12_: $GW\;|\equiv SN\overset{{TC}_{j}}{↔}\;GW$
A_13_: $GW\;|\equiv SN\overset{k_{i}}{↔}\;GW$
A_14_: SN |≡⋕(TS_2_)
A_15_: SN |≡⋕(TS_3_)
A_16_: $SN\;|\equiv SN\overset{{TC}_{j}}{↔}\;GW$
A_17_: $SN\;|\equiv SN\overset{k_{i}}{↔}\;GW$
A_18_: $SN\;|\equiv GW\Rightarrow U\overset{SK}{↔}\;SN$
A_19_: $SN\;|\equiv GW\Rightarrow U\overset{k_{i}}{↔}\;SN$
A_20_: $SN\;|\equiv U\Rightarrow U\overset{SK}{↔}\;SN$
A_21_: $SN\;|\equiv U\Rightarrow U\overset{k_{i}}{↔}\;SN$

**Second**, our scheme is transformed to the idealized form.

M_1_: $\text{U}\to \text{GW}:{\left({\text{ TS}}_{1},\text{U}\overset{{\text{k}}_{\text{i}}}{↔}\text{ SN}\right)}_{{\text{TC}}_{\text{i}}}$
M_2_: $\text{GW}\to \text{SN}:{\left({\text{ TS}}_{2},\text{U }|\equiv \text{U}\overset{{\text{k}}_{\text{i}}}{↔}\text{ SN}\right)}_{{\text{TC}}_{\text{j}}}$
M_3_: $\text{SN}\to \text{U}:{\left({\text{ TS}}_{3},\text{U}\overset{{\text{k}}_{\text{i}}}{↔}\text{ SN},\text{U}\overset{{\text{k}}_{\text{j}}}{↔}\text{ SN}\right)}_{{\text{k}}_{\text{i}}}$

**Third**, the idealized form of our scheme is analyzed based on BAN-logic and the assumptions. The main steps are described as follows:

By M_1_ and the seeing rule, we get:
  S_1_: $GW⊲\;{\;({TS}_{1},U\overset{k_{i}}{↔}\;SN)}_{{TC}_{i}}$
By A_10_, S_1_ and the message-meaning rule, we get:
  S_2_: $GW\;|\equiv U\;|∼\;({TS}_{1},U\overset{k_{i}}{↔}\;SN)$
By A_8_, S_2_, freshness rule and nonce-verification, we get:
  S_3_: $GW\;|\equiv U\;|\equiv \;U\overset{k_{i}}{↔}\;SN$
By M_2_ and the seeing rule, we get:
  S_4_: $SN⊲\;{\;({TS}_{2},U\;|\equiv U\overset{k_{i}}{↔}\;SN)}_{{TC}_{j}}$
By A_16_, S_4_ and the message-meaning rule, we get:
  S_5_: $SN\;|\equiv GW\;|∼\;({TS}_{1},U\;|\equiv U\overset{k_{i}}{↔}\;SN)$
By A_14_, S_5_, freshness rule and nonce-verification, we get:
  S_6_: $SN\;|\equiv GW\;|\equiv (U\;|\equiv \;U\overset{k_{i}}{↔}\;SN)$
By A_19_, S_6_ and the jurisdiction rule, we get:
  S_7_: $\text{SN }\left|\equiv \text{U }\right|\equiv \text{ U}\overset{{\text{k}}_{\text{i}}}{↔}\text{ SN}\;\;\;\;\left(\text{Goal }8\right)$
By A_21_, S_7_ and the jurisdiction rule, we get:
  S_8_: $\text{SN }|\equiv \text{ U}\overset{{\text{k}}_{\text{i}}}{↔}\text{ SN}\;\;\;\;\left(\text{Goal }7\right)$
By S_7_ and session key rule which k_i_ is the necessary parameters of SK, we get:
  S_9_: $\text{SN }\left|\equiv \text{U }\right|\equiv \left(\text{ U}\overset{\text{SK}}{↔}\text{ SN}\right)\;\;\;\left(\text{Goal }4\right)$
By A_20_, S_9_ and the jurisdiction rule, we get:
  S_10_: $\text{SN }|\equiv \text{ U}\overset{\text{SK}}{↔}\text{ SN}\;\;\left(\text{Goal }3\right)$
By M_3_ and the seeing rule, we get:
  S_11_: $U⊲\;{\;({TS}_{3},U\overset{k_{i}}{↔}\;SN,U\overset{k_{j}}{↔}\;SN)}_{k_{i}}$
By A_10_, S_11_ and the message-meaning rule, we get:
  S_12_: $U\;|\equiv SN\;|∼\;({TS}_{3},U\overset{k_{i}}{↔}\;SN,U\overset{k_{j}}{↔}\;SN)$
By A_15_, S_12_ freshness rule and nonce-verification, we get:
  S_13_: $U\;|\equiv SN\;|\equiv (U\overset{k_{i}}{↔}\;SN,U\overset{k_{j}}{↔}\;SN)$
By S_13_ and the belief rule, we get:
  S_14_: $\text{U }\left|\equiv \text{SN }\right|\equiv \text{ U}\overset{{\text{k}}_{\text{i}}}{↔}\text{ SN}\;\;\left(\text{Goal }6\right)$
  S_15_: $U\;|\equiv SN\;|\equiv \;U\overset{k_{j}}{↔}\;SN$
By A_6_, S_14_ and the jurisdiction rule, we get:
  S_16_: $\text{U }|\equiv \text{ U}\overset{{\text{k}}_{\text{i}}}{↔}\text{ SN}\;\;\left(\text{Goal }5\right)$
By S_15_ and the session key rule which k_j_ is the necessary parameters of SK, we get:
  S_17_: $\text{U }\left|\equiv \text{SN }\right|\equiv \left(\text{ U}\overset{\text{SK}}{↔}\text{ SN}\right)\;\;\left(\text{Goal }2\right)$
By A_5_, S_17_ and the jurisdiction rule, we get:
  S_18_: $\text{U }|\equiv \text{ U}\overset{\text{SK}}{↔}\text{ SN}\;\;\left(\text{Goal }1\right)$

From the above discussion, our scheme satisfies (Goal 1), (Goal 2), (Goal 3), (Goal 4), (Goal 5), (Goal 6), (Goal 7) and (Goal 8). Therefore, U, GW and SN perform the mutual authentication and session key exchange securely.

### Informal analysis

In this section, we prove our scheme could withstand other attacks. The detailed analysis is described as follows.

#### Stolen smart card attacks

We know that A could use a power analysis attack to extract the information stored in the SC. We assume that A obtains information (e_i_, V_i_, PTC_i_, ID_SC_). These messages are operated after a one-way hash function. The multiple passwords and the secret number K_i_ from the SC are impossible to obtain. Because A meets the property of the one-way hash function [48–50], our scheme can withstand the stolen SC attacks.

#### Nodes captured attacks

After WSNs are deployed in the target field, A can easily capture a legitimate sensor node [59–61]. Although there are some important studies that focus on the key revocation protocols [63, 64], we believe the confidentiality of stored key/data is as important as key revocation. We assume that A could obtain (PTC_j_, PK_j_) from SN. Owing to the properties of the one-way hash function and XOR operation [51–53], the secret number k_j_ or TC_j_ are impossible to obtain from SN. Given that ID_SN_ is replaced with PID_j_ in the registration phase, A cannot extract ID_SN_. The secret number, k_j_, is impossible to guess because of the two unknown numbers. To obtain TC_j_, A can compute TC_j_ = PTC_j_⨁PID_j_. However, PID_j_ is not stored in SN. Therefore, A cannot obtain TC_j_. According to the preceding discussion, we can conclude that our proposed scheme can withstand the nodes captured attack.

#### Privileged insider attacks

We assume that the adversary A is a privileged insider of WSNs. Therefore, A can access GW to obtain others’ sensitive information. In our scheme, GW does not store the passwords of U and other sensitive information. Therefore, A cannot extract the passwords of U. We assume that A can obtain (PK_GW_, PID_j_) from GW. Given the properties of the one-way hash function and XOR operation, deriving k_GW_ and TC_i_ is an almost impossible task for A. We assume that A intends to compute k_GW_ = PTC_i_⨁PK_GW_. However, since PTC_i_ is stored in the SC of U, A cannot obtain k_GW_. The preceding discussion shows that our proposed scheme can withstand privileged insider attacks.

#### Impersonation attacks/ mutual authentication

The adversary A can impersonate the GW to send/receive the message or install any program to take over the entire network [65]. In our scheme, each receiver must authenticate the identity of the sender by MAC and Ver functions with the sender’s own secret key. GW verifies the identity of U by computing ${Ver}_{k_{i}}({TC}_{i}∥{TS}_{1}∥{RPW}_{i},C_{i})$ = 1? with K_i_. SN verifies the identity of GW by ${Ver}_{{TC}_{j}}(k_{i}∥{TS}_{2}∥{PID}_{j},C_{GW})$ = 1? with TC_j_. U verifies the identity of SN by ${Ver}_{k_{j}}(k_{j}∥{TS}_{3}∥k_{i},C_{j})$ = 1? with K_j_. A cannot impersonate any legitimate entity without knowing the secret numbers, such as K_i_, TC_j_, and k_j_. Accordingly our proposed scheme can withstand an impersonation attack and achieve mutual authentication.

#### User anonymity

According to Choo et al.’ research [66], there is a mechanical approach to derive identity-based schemes from existing Diffie-Hellman-based schemes. After a careful study of this work, our scheme is designed to withstand this method for protecting user’s anonymity. In the login phase, our proposed scheme uses ID_SC_ as the only identity of U. However, a serious problem with user privacy exists. User anonymity is necessary to resist tracing attacks. Our scheme hides ID_SC_ in RPW_i_ = H(ID_SC_ ∥ PW_1_ ∥ PW_2_ ∥ ⋯∥PW_n_ ∥ n ∥ k_i_), V_i_ = H(e_i_ ∥ RPW_i_ ∥ ID_SC_ ∥ k_i_ ∥n) and DID_SC_ = ID_SC_⊕H(TS_1_ ∥ ID_GW_). The transmitted pseudo identity DID_SC_ is the dynamic name. Given the hash function property, A cannot extract ID_SC_ without ID_GW_. Consequently, our scheme achieves the goal of anonymity and can withstand tracing attacks.

#### Online guessing attacks

In our scheme, the registration phase is executed strictly in a secure environment before deployment. We assume that A intercepts message transmission in the channel during the login, authentication and key exchange, password updating, and dynamic-node addition phases. A can obtain the messages (PTC_i_, C_i_, PKS_i_, TS_1_), (PID_j_, C_GW_, PKS_GW_, TS_2_), and (C_j_, PKS_j_, TS_3_), (${PTC}_{i}\;{PTC}_{i}^{new}$). Notably, the intercepted message, excluding the TS, is entirely encrypted by hash function and XOR operation. In addition, each hash function includes a minimum of two unknown numbers. Therefore, A cannot use online guessing attacks to guess the inputs of the hash function. In C_GW_ and ${Ver}_{{TC}_{j}}$ calculation, although only one unknown input is in the function, A cannot guess the inputs from the dictionary without the secret key, TC_j_. Therefore, our scheme can resist online guessing attacks.

#### Offline password guessing attacks

Offline password guessing attacks have always been a major security concern in designing password-based schemes. There are some outstanding studies trying to solve this problem, and our scheme strictly observes the rules that are described in Nam et al.’s research [67]. In this attack analysis section, A can use the power analysis attack to extract the information stored in the SC. Therefore, A obtains (e_i_, V_i_, PTC_i_, ID_SC_) from SC. All messages extracted by A are operated by hash function and XOR operation. Therefore, A cannot derive the sensitive information from these messages. Each message includes a minimum of two unknown inputs, as well as multiple passwords encrypted by the hash function. Therefore, A cannot use offline password-guessing attacks to derive the multiple passwords and the number of passwords n from the SC.

#### Replay attacks

We assume that A intercepts the messages transmitted in the communication channel and replays these messages to the receiver without any modification. A replay attack cannot work in our scheme because each entity initially checks the freshness of the TS. If the TS is not fresh, then the receiver rejects the request. Therefore, our scheme can resist replay attacks.

#### Man-in-the-middle attacks

Choo et al. proposed that the unknown key share attack(man-in-the-middle attack) is the most fatal security problem for any protocol [68]. We assume that A intercepts the messages transmitted in the communication channel and replays these messages to the receiver with a particular modification of the message. The purpose of this action of A  is to make the receiver believe that A is the legitimate sender. A can intercept the transmitted messages via the channel. To pass authentication, A must compute C_i_, C_GW_, and C_j_ and A is unable to obtain (TC_i_, RPW_i_, k_i_, TC_j_, k_j_) without knowing the secret number or the temporal credential of each entity. Therefore, A cannot obtain the right (C_i_, C_GW_, C_j_) and pass authentication. Therefore, our scheme can resist man-in-the-middle attacks.

#### Lost password threat

According to other studies [69–71], passwords are currently not safe and are therefore vulnerable to any identity authentication. A can obtain the used passwords of U through numerous methods. For example, A can obtain user passwords from a low-security level database or by using social engineer [44, 45]. Then, A can use these lost passwords to pass the authentication of WSNs with the stolen SC. Once the password is lost, the scheme for WSNs encounters a considerable threat. In our scheme, multiple passwords are used to replace the unique password, which means that the legitimate user needs to input several passwords at will. The passwords, their sequence, and their number are used as key factors to authenticate the user’s identity. Although A obtains the used passwords, he/she does not know other security factors, such as the sequence of passwords, their combination, and their number. In other schemes, if A obtains *m* passwords of the user, the probability of obtaining the correct password is described as follows:

$$P_{\text{one}}=\frac{1}{m}\times P_{\text{h}},$$

where we assume that the probability of using the old password is *P*_h_. In our scheme, U adopts *n* passwords as login passwords. The probability of obtaining the correct password is

$$P_{\text{multiple}}=\frac{1}{A_{\text{m}}^{\text{n}}}\times \frac{1}{m}\times P_{\text{h}}.$$

If the lost passwords do not consist of all the multiple passwords, the probability is smaller than *P*_multiple_. According to the preceding discussion, *P*_multiple_ is smaller than *P*_one_, and A cannot obtain the correct multiple passwords. Therefore, our scheme can prevent the lost password threat.

#### D-DOS attacks

Because of the energy limitation of WSNs, D-DOS attack is one of the most detrimental threats to WSNs [3, 42, 59], this attack includes the hello flood, inputting the wrong password, and resource depletion attacks. The goal of these attacks is to deplete the resource, especially the energy of WSNs. Numerous related schemes verify the user identity in GW with several complex computations, including numerous hash functions and other operations. This authentication method costs considerable energy of WSNs if A starts a D-DOS attack, which is launched by persistently inputting wrong passwords persistently. Our scheme verifies the user identity by the SC without any consumption of GW. This idea can cut the spare overhead off and can validly resist the D-DOS attacks that are launched by inputting wrong passwords in the login phase.

#### Malicious sensor-node attack

In the dynamic-node addition phase, U can add his/her new SNs to the WSNs. If the SN^new^ is the malicious sensor node that is employed by A, then SN^new^ can obtain information from other legitimate SNs and start malicious sensor-node attacks on WSNs, including Sybil, wormhole, sink hole, rushing, routing loop, and other types of attacks [1, 40]. To protect WSNs from malicious sensor-node attacks, our scheme requires the procedure of the dynamic node addition phase to be executed under the legitimate user. If someone wants to add any new SN to the WSN, the validity of the user identity must be verified. If the identity is not legitimate, the request is rejected. Therefore, our scheme can withstand malicious sensor-node attacks.

#### Three-factor security

Numerous related schemes adopting three security factors [20, 72, 73] usually adopt SC, password, and biometric characteristics as authenticating factors. However, biometrics present several drawbacks that are unsuitable for WSNs. Therefore, our scheme uses multiple passwords to replace the biometric characteristic. Several passwords, their sequence, and the number of passwords are used as the most important factors for verification.

#### Integrity of message

In our scheme, the MAC and Ver functions are used to achieve the goal of confidentiality and integrity, which are the most important properties of security [74, 75]. Upon receiving messages, the receiver verifies whether the output of the Ver function is equal to 1. If it is not equal, the receiver aborts the session and rejects the request from the sender. Therefore, if A modifies the message and sends it to the next entity, then the message is denied. Therefore, our scheme checks the integrity of the message.

### Security performance comparison

In this section, we compare our proposed scheme with other schemes from the security aspect. The comparison shows that our scheme exhibits superior security performance to other schemes. The detailed comparison is presented in Table 1. Yes and No in this table denote that the scheme could withstand the attack or could not withstand the attack, respectively, and n/a denotes the scheme is not applicable in this comparison. The abbreviations below Table 1 denote the compared security properties [76].

## Performance Analysis

In this section, we compare our proposed scheme with other schemes that are listed in Table 1. As introduced in other studies [6, 72], the overhead of several base operations, such as XOR operation, TS, and random number generation are ignored. These types of operations entail approximately no cost in comparison with the one-way hash computation and other complex computations. We believe that the communication overhead and storage overhead are of equal importance to the computational overhead. As introduced in Amin et al.’s research [76], the communication and storage overheads are analyzed in detail. Therefore, we analyze our scheme in three terms.

### Reference basis

In this section, we enumerate the reference basis of WSN performance that is adopted in this paper. As described in several studies [14, 17–21, 23, 35, 73, 77–83], all protocols are compared by the number of main computations. To show the result intuitively, we unified the hash function to represent all protocol overheads. The basis of comparison is described as follows:

1. According to Nam et al.’s research [14] and Crypto++ 5.6.0 benchmarks, we know that SHA-1 takes 11.4 cycles per byte, HMAC takes 11.9 cycles per byte, and AES takes 16.9 cycles per byte under Windows Vista and Intel Core 2. Therefore, one HMAC is equal to 1.04 hash functions and one AES is almost equal to 1.5 hash functions.
2. As introduced in other studies [72, 84], one asymmetric encryption/decryption is equal to 100 symmetric encryptions/decryptions. In addition, a symmetric encryption/decryption is at least 60 times faster than a one-exponential operation.
3. According to other studies [20, 39, 72], the time to execute a fuzzy extractor is the same as for an elliptic curve point multiplication. The time for a one-way hashing operation is 0.00032 s, for a symmetric encryption/decryption operation is 0.0056 s, for a modular exponentiation operation is 0.0192 s, and for an elliptic curve point relative multiplication operation or a fuzzy extractor is 0.0171 s.
4. According to Ma’s study [85], we assume one WSN that adopts MICA2 and, integrates an 8 bit 8 MHz ATmega128L processor with the voltage is 3 V, the computational electric current is 8 mA, the received electric current is 10 mA, the transmitted electric current is 27 mA, and the transmission rate is 12.4 kb/s. Therefore, the executed 0.00032 s computation needs 3 V × 8 mA × 0.00032 s = 0.00768 mJ.
5. In agreement to [6, 20], we assume that the hash output is 160 bits [86], one prime factor is 160 bits minimum, the elliptical curve output is 320 bits, and the secret parameter is at least 160 bits [87]. The TS has 32 bits; expiration time for TE, is 32 bits; the user identity ID, pseudo ID, and random nonce are 160 bits; sensor node identity ID_SN_, GW ID_GW_, and pseudo ID_SN_ are 16 bits; encryption/decryption output is 128 bits; MAC output is 128 bits; and key setup is 128 bits.

Therefore, we can conclude all main computations in several aspects. The overhead of these main computations is described in Table 2.

**Table 2:** The comparison with main computations.

	cycles per byte	hash function	the time(s)	consumption(mJ)
*T*_*H*_	11.4	1 *T*_*H*_	0.00032	0.00768
*T*_*A*_	1140	about 150 *T*_*H*_	0.048	1.152
*T*_*E*_	16.9	about 1.5 *T*_*H*_	0.00048	0.01152
*T*_*M*_	11.9	about 1 *T*_*H*_	0.00032	0.00768
*T*_*ME*_	684	about 60 *T*_*H*_	0.0192	0.4608
*T*_*Ex*_	1026	about 90 *T*_*H*_	0.336	8.064
*T*_*EC*_/*T*_*F*_	609	about 53 *T*_*H*_	0.0171	0.4104

The notations in this section are as follows:

*T*_*H*_: hash function operation;*T*_*A*_: asymmetric encryption/decryption; *T*_*E*_: symmetric encryption/decryption;*T*_*M*_: MAC generation/verification;*T*_*ME*_: modular exponentiation operation;*T*_*Ex*_: one-exponential operation; *T*_*EC*_: elliptic curve point multiplication;*T*_*F*_: fuzzy extractor.

#### Comparison with other schemes

In this section, we compare our proposed scheme with the schemes proposed by Nam et al. [14], A. K. Das [20], He et al. [21], Jiang et al. [19], M. L. Das [17], and Xue et al. [18] in terms of computational, communication, and storage overheads. Comparison details are described as follows.

#### Computational overhead

In this section, we compare the computational overhead of all schemes in several aspects. The details of the comparison of computational overhead are shown in Table 3. Notation: the numbers shown in Table 3 is a rough number that retains three decimal places.

**Table 3:** Comparison of computational overhead.

Phase	Login		Authentication and key agreement			Total	hash	time(s)	enegy (mJ)
	U	GW	U	GW	SN				
Nam et al.	2*T*_*EC*_ + 2*T*_*H*_ + 1*T*_*E*_ + 1*T*_*M*_	1*T*_*EC*_ + 1*T*_*H*_ + 2*T*_*E*_ + 1*T*_*M*_	2*T*_*H*_	1*T*_*E*_ + 1*T*_*M*_	1*T*_*M*_ + 2*T*_*H*_ + 1*T*_*E*_	3*T*_*EC*_ + 7*T*_*H*_ + 5*T*_*E*_ + 4*T*_*M*_	177.5*T*_*H*_	0.0568	1.363
A.K.Das	1*T*_*F*_ + 3*T*_*H*_	0	6*T*_*H*_	11*T*_*H*_	5*T*_*H*_	1*T*_*F*_ + 25*T*_*H*_	78*T*_*H*_	0.0251	0.602
He et al.	5*T*_*H*_	4*T*_*H*_	3*T*_*H*_	5*T*_*H*_	6*T*_*H*_	23*T*_*H*_	23*T*_*H*_	0.00736	0.177
Jiang et al.	3*T*_*H*_	2*T*_*H*_	4*T*_*H*_	7*T*_*H*_	5*T*_*H*_	21*T*_*H*_	21*T*_*H*_	0.00672	0.161
M.L.Das	4*T*_*H*_	0	0	4*T*_*H*_	1*T*_*H*_	9*T*_*H*_	9*T*_*H*_	0.00288	0.054
XUE etal.	2*T*_*H*_	0	8*T*_*H*_	11*T*_*H*_	6*T*_*H*_	27*T*_*H*_	27*T*_*H*_	0.00864	0.207
**Ours**	3*T*_*H*_	0	4*T*_*H*_ + 2*T*_*M*_	5*T*_*H*_ + 2*T*_*M*_	3*T*_*H*_ + 2*T*_*M*_	15*T*_*H*_ + 6*T*_*M*_	21*T*_*H*_	0.00672	0.161

#### Communication overhead

As introduced by the study [6], the transmission overhead is considerably larger than the computational overhead. The proportion of all overheads is listed as follows: 71% data transmission, 20% MAC transmission, 7% nonce transmission (for freshness), and 2% MAC and encryption computation. Therefore, analyzing the communication overhead is crucial. We assume that the receiving electric current of WSNs is 10 mA, the transmitting electric current is 27 mA, and the rate of transmission is 12.4 kb/s. According to Ma’s study [85], we assume that 1-byte transmission consumption is 3 V × 27 mA × 8 b/12400 b/s = 0.052mJ and a received byte consumption is 3 V × 10 mA × 8 b/12400 b/s = 0.019 mJ.

The details of the communication overhead of all schemes are presented in Table 4. The hello and successful signals are ignored. Notation: the number shown in Table 4 is a rough number that retains three decimal places.

**Table 4:** Comparison of communication overhead.

schemes	Total bits	Rough consumption(mJ)			
		U	GW	SN	total
Nam et al.	1264	4.463	4.645	2.206	11.314
A.K.Das	1952	4.7	9.132	3.643	17.475
He et al.	1744	5.489	6.093	4.03	15.612
Jiang et al.	1920	4.622	8.923	3.643	17.188
M.L.Das	704	2.299	3.151	0.852	6.302
XUE et al.	1744	5.489	6.093	4.03	15.612
**Ours**	1440	4.955	4.683	3.251	12.899

#### Storage overhead analysis

In this section, we compare the size of stored messages with other schemes. According to the reference basis, we compute the size of stored messages in U, GW, and SN, respectively. The detailed comparison of storage overhead is presented in Table 5.

**Table 5:** Comparison of storage overhead.

schemes	The storage overhead			
	U/SC	GW	SN	Total (bit)
Nam et al.	*P*, *XEID*_*U*_, *Y*, *ID*_*GW*_	*y*, *EID*_*U*_, *Y*, *ID*_*GW*_, *k*_*GS*_	*ID*_*SN*_, *k*_*GS*_	1648
A.K.Das	$r_{i}^{*},f_{i},e_{i},TID_{i},TE_{i},PTC_{i}$	*TID*_*i*_, *X*_*S*_, *K*_*GWN−S*_, *TE*_*i*_, *ID*_*i*_, *ID*_*SN*_	*TC*_*j*_, *ID*_*SN*_	3424
He et al.	*r*_*i*_, *PID*_*i*_, *TE*_*i*_, *PTC*_*i*_	*SID*_*j*,_ *H(PW*_*j*_), *K*_*GWN−S*,_ *K*_*GWN−U*_	*TC*_*j*_, *ID*_*SN*_	1184
Jiang et al.	r, *TID*_*i*_, *TE*_*i*,_ *PTC*_*i*_	*TID*_*i*_, *TE*_*i*_, *ID*_*i*_, *K*_*GWN−S*_, *ID*_*SN*_	*TC*_*j*_, *ID*_*SN*_	2080
M.L.Das	*ID*_*i*_, *H*(*PW*_*i*_), *N*_*i*_, *x*_*a*_	*ID*_*i*_, *K*, *N*_*i*_, *x*_*a*_, *S*_*N*_	*S*_*N*_, *x*_*a*_	1472
XUE et al.	*ID*_*i*_, H(H(*PW*_*i*_)), *TE*_*i*_, *PTC*_*i*_	*K*_*GWN*−*S*,_ *K*_*GWN*−*U*_, *ID*_*SN*_	*TC*_*j*_, *ID*_*SN*_	1024
Ours	*PTC*_*i*_, *V*_*i*_, *e*_*i*_, *ID*_*GW*_,	*PK*_*GW*_, *PID*_*j*_, *ID*_*GW*_, *ID*_*SC*_	*PTC*_*J*_, *PK*_*j*_	1328

#### Comparison of total overhead

In this section, we compare the total overhead of schemes, including communication and computation overheads. We compare the overhead of each entity in Table 6 and compute the total overhead of all schemes. The result shows that the communication consumption is markedly larger than the computation consumption and the percentage is almost above 95% of the total overhead, and the result is the same as that in Perrig et al.’s study [6] and in common agreement with other research. Future security schemes developed will be compared based on computation overhead and communication overhead. Owing to the property of WSNs [85], the gateway station presents larger energy, higher computation performance, and larger storage performance than SN. If we want to improve the overhead of the research scheme, the most important point is improving the communication overhead of SN instead of computational overhead. Notation: the number shown in Table 6 is a rough number that retains three decimal places. The notations in this section are denoted as follows: CC: communication costs; PC: computation costs; Tot: total overhead %: the communication costs’ percentage of total overhead.

**Table 6:** Comparison of total consumption.

schemes	Rough total consumption(mJ)										
	U			GW			SN			total	%
	CC	PC	Tot	CC	PC	Tot	CC	PC	Tot		
Nam et al.	4.463	0.864	5.327	4.645	0.465	5.11	2.206	0.035	2.241	12.678	89.24
A.K.Das	4.7	0.476	5.176	9.132	0.084	9.216	3.643	0.038	3.681	18.073	96.69
He et al.	5.489	0.061	5.55	6.093	0.069	6.162	4.03	0.046	4.076	15.788	98.89
Jiang et al.	4.622	0.054	4.676	8.923	0.069	8.992	3.643	0.038	3.681	17.349	99.07
M.L.Das	2.299	0.03	2.329	3.151	0.03	3.181	0.852	0.008	0.86	6.37	98.93
XUE et al.	5.489	0.077	5.566	6.093	0.084	6.177	4.03	0.046	4.076	15.819	98.69
Ours	4.955	0.069	5.024	4.883	0.054	4.937	3.251	0.038	3.289	13.25	98.41

## Conclusion

In this paper, we designed a temporal credential-based mutual authentication with a multiple-password scheme for WSNs. Through comparison with other schemes, we have proven that our scheme exhibits better security performance than the other schemes. Moreover, our scheme can withstand related attacks, including the lost password threat. The discussion in this paper proves that our scheme entails relatively small consumption. The analysis shows that the communication consumption’s percentage of total overhead is almost above 95% and it is markedly larger than the computational consumption. Therefore, we will compare future security schemes based on computational overhead and communication overhead.

## Supporting Information

S1 Table The security comparison with other schemes. This table illustrates the security comparison with other schemes. The comparison show that our scheme has better security performance than others.
(DOCX)

S2 Table The comparison with main computations. This table illustrates the main computations in the authentication scheme for wireless sensor networks.
(DOCX)

S3 Table Comparison of computational overhead. This table illustrates the computational overhead comparison with other schemes. The comparison shows that our scheme has better performance than others in computational overhead.
(DOCX)

S4 Table Comparison of communication overhead. This table illustrates the communication overhead comparison with other schemes. The comparison shows that our scheme has better performance than others in communication overhead.
(DOCX)

S5 Table Comparison of storage overhead. This table illustrates the storage overhead comparison with other schemes. The comparison shows thatour scheme has better performance than others in storage overhead.
(DOCX)

S6 Table Comparison of total consumption. This table illustrates the comparison with other schemes. The detailed comparison shows that the communication overhead accounts for the majority of total overhead.
(DOCX)
