# Supplementary material for: A Temporal Credential-Based Mutual Authentication with Multiple-Password Scheme for Wireless Sensor Networks
Source: PLoS One. 2017 Jan 30;12(1):e0170657. doi: 10.1371/journal.pone.0170657 (PMC5279753; doi:10.1371/journal.pone.0170657)
Supplement: S1 Table — This table illustrates the security comparison with other schemes. The comparison show that our scheme has better security performance than others. (DOCX) [file pone.0170657.s001.docx]

**Table 1.The security comparison with other schemes**

|  | SSCA | NCA | PIA | MA | UA | ONGA | OFPGA | RA | MITMA | LPT | DDA | MSNA | TFS | IOM | IGA | SKA | PUP | DNAP |
| --- | --- | --- | --- | --- | --- | --- | --- | --- | --- | --- | --- | --- | --- | --- | --- | --- | --- | --- |
| D.B.He | yes | no | no | yes | yes | no | yes | yes | yes | no | no | n/a | no | yes | yes | yes | no | no |
| A.K.Das | yes | no | yes | yes | yes | yes | yes | yes | yes | no | yes | no | yes | no | yes | yes | yes | yes |
| J.H.Nam | no | yes | yes | no | yes | no | no | no | no | no | no | n/a | no | yes | no | yes | yes | no |
| K.XUE | no | no | no | yes | no | yes | no | yes | yes | no | no | n/a | no | no | no | yes | yes | no |
| Q.Jiang | no | no | no | no | yes | no | yes | yes | yes | no | no | n/a | no | yes | no | yes | no | no |
| M.L.Das | no | no | no | yes | no | no | yes | yes | yes | no | no | n/a | no | no | no | no | no | no |
| Ours | yes | yes | yes | yes | yes | yes | yes | yes | yes | yes | yes | yes | yes | yes | yes | yes | yes | yes |

SSCA: Stolen smart card attack; NCA: Nodes captured attack; PIA: Privileged insider attack; MA: mutual authentication; UA: Anonymity; ONGA: Online guessing attack OFPGA: Off-line password guessing attack; RA: Replay attack; MITMA: Man-in-the-middle attack; LPT: Lost password threat; DDA: D-Dos attack; MSNA: Malicious sensor node attacks; TFS: Three-factor security; IOM: Integrity of message; IGA: identity guessing attack; SKA: session key agreement; SKA: session key agreement; PUP: password updated phase; DNAP: dynamic node addition phase
