# Supplementary material for: A Temporal Credential-Based Mutual Authentication with Multiple-Password Scheme for Wireless Sensor Networks
Source: PLoS One. 2017 Jan 30;12(1):e0170657. doi: 10.1371/journal.pone.0170657 (PMC5279753; doi:10.1371/journal.pone.0170657)
Supplement: S2 Table — This table illustrates the main computations in the authentication scheme for wireless sensor networks. (DOCX) [file pone.0170657.s002.docx]

**Table 2 the comparison with main computations**

|  | cycles per byte | hash function | the time(s) | consumption(mJ) |
| --- | --- | --- | --- | --- |
| $T_{H}$ | 11.4 | 1 $T_{H}$ | 0.00032 | $0.00768$ |
| $T_{A}$ | 1140 | about 150$T_{H}$ | 0.048 | $1.152$ |
| $T_{E}$ | 16.9 | about 1.5 $T_{H}$ | 0.00048 | $0.01152$ |
| $T_{M}$ | 11.9 | about 1 $T_{H}$ | 0.00032 | $0.00768$ |
| $T_{ME}$ | 684 | about 60 $T_{H}$ | 0.0192 | $0.4608$ |
| $T_{Ex}$ | 1026 | about 90 $T_{H}$ | 0.336 | $8.064$ |
| $T_{EC}/T_{F}$ | 609 | about 53$T_{H}$ | 0.0171 | $0.4104$ |
