# Supplementary material for: A Temporal Credential-Based Mutual Authentication with Multiple-Password Scheme for Wireless Sensor Networks
Source: PLoS One. 2017 Jan 30;12(1):e0170657. doi: 10.1371/journal.pone.0170657 (PMC5279753; doi:10.1371/journal.pone.0170657)
Supplement: S3 Table — This table illustrates the computational overhead comparison with other schemes. The comparison shows that our scheme has better performance than others in computational overhead. (DOCX) [file pone.0170657.s003.docx]

**Table 3 the comparison of computational overhead**

| Phase | Login | | Authentication and key agreement | | | Total | hash | time(s) | enegy (mJ) |
| --- | --- | --- | --- | --- | --- | --- | --- | --- | --- |
|  | $U$ | $\mathrm{GW}$ | $U$ | $\mathrm{GW}$ | $\mathrm{SN}$ |  |  |  |  |
| Nam et al. | $2T_{EC}+2T_{H}+1T_{E}+1T_{M}$ | $1T_{EC}+1T_{H}+2T_{E}+1T_{M}$ | $2T_{H}$ | $1T_{E}+$  $1T_{M}$ | $1T_{M}+2T_{H}+1T_{E}$ | $3T_{EC}+7T_{H}+5T_{E}+4T_{M}$ | $177.5T_{H}$ | 0.0568 | 1.363 |
| A.K.Das | ${1T}_{F}+3T_{H}$ | 0 | $6T_{H}$ | $11T_{H}$ | $5T_{H}$ | ${1T}_{F}+25T_{H}$ | $78T_{H}$ | 0.0251 | 0.602 |
| He et al. | $5T_{H}$ | $4T_{H}$ | $3T_{H}$ | $5T_{H}$ | $6T_{H}$ | $23T_{H}$ | $23T_{H}$ | 0.00736 | 0.177 |
| Jiang et al. | ${3T}_{H}$ | ${2T}_{H}$ | ${4T}_{H}$ | ${7T}_{H}$ | ${5T}_{H}$ | ${21T}_{H}$ | ${21T}_{H}$ | 0.00672 | 0.161 |
| M.L.Das | ${4T}_{H}$ | 0 | 0 | ${4T}_{H}$ | ${1T}_{H}$ | $9T_{H}$ | $9T_{H}$ | 0.00288 | 0.054 |
| XUE et al. | ${2T}_{H}$ | 0 | $8T_{H}$ | ${11T}_{H}$ | ${6T}_{H}$ | ${27T}_{H}$ | ${27T}_{H}$ | 0.00864 | 0.207 |
| **Ours** | ${3T}_{H}$ | 0 | ${4T}_{H}+2T_{M}$ | ${5T}_{H}+2T_{M}$ | ${3T}_{H}+2T_{M}$ | ${15T}_{H}+6T_{M}$ | ${21T}_{H}$ | 0.00672 | 0.161 |
