# Supplementary material for: A Temporal Credential-Based Mutual Authentication with Multiple-Password Scheme for Wireless Sensor Networks
Source: PLoS One. 2017 Jan 30;12(1):e0170657. doi: 10.1371/journal.pone.0170657 (PMC5279753; doi:10.1371/journal.pone.0170657)
Supplement: S4 Table — This table illustrates the communication overhead comparison with other schemes. The comparison shows that our scheme has better performance than others in communication overhead. (DOCX) [file pone.0170657.s004.docx]

**Table 4. Comparison of communication overhead**

| schemes | Total bits | Rough consumption($\mathrm{mJ}$) | | | |
| --- | --- | --- | --- | --- | --- |
|  |  | $U$ | $\mathrm{GW}$ | $\mathrm{SN}$ | total |
| Nam et al. | 1264 | 4.463 | 4.645 | 2.206 | 11.314 |
| A.K.Das | 1952 | 4.7 | 9.132 | 3.643 | 17.475 |
| He et al. | 1744 | 5.489 | 6.093 | 4.03 | 15.612 |
| Jiang et al. | 1920 | 4.622 | 8.923 | 3.643 | 17.188 |
| M.L.Das | 704 | 2.299 | 3.151 | 0.852 | 6.302 |
| XUE et al. | 1744 | 5.489 | 6.093 | 4.03 | 15.612 |
| **Ours** | 1440 | 4.955 | 4.683 | 3.251 | 12.899 |
