# Supplementary material for: A Temporal Credential-Based Mutual Authentication with Multiple-Password Scheme for Wireless Sensor Networks
Source: PLoS One. 2017 Jan 30;12(1):e0170657. doi: 10.1371/journal.pone.0170657 (PMC5279753; doi:10.1371/journal.pone.0170657)
Supplement: S5 Table — This table illustrates the storage overhead comparison with other schemes. The comparison shows thatour scheme has better performance than others in storage overhead. (DOCX) [file pone.0170657.s005.docx]

**Table 5. Comparison of storage overhead**

| The storage overhead | | | | |
| --- | --- | --- | --- | --- |
| schemes | $U/SC$ | $\mathrm{GW}$ | $\mathrm{SN}$ | Total  (bit) |
| Nam et al. | $P,{XEID}_{U},Y,{ID}_{GW}$ | $y,{EID}_{U},Y,{ID}_{GW},k_{GS}$ | ${ID}_{SN},k_{GS}$ | 1648 |
| A.K.Das | $r_{i}^{*},f_{i},e_{i},{TID}_{i},{TE}_{i},{PTC}_{i}$ | ${TID}_{i},X_{S},K_{GWN-S}$,${TE}_{i},{ID}_{i},{ID}_{SN}$ | ${TC}_{j},{ID}_{SN}$ | 3424 |
| He et al. | $r_{i},{PID}_{i},{TE}_{i}, {PTC}_{i}$ | ${SID}_{j},{H(PW}_{j}),K_{GWN-S},K_{GWN-U}$ | ${TC}_{j},{ID}_{SN}$ | 1184 |
| Jiang et al. | $r,{TID}_{i},{TE}_{i},{PTC}_{i}$ | ${TID}_{i},{TE}_{i},{ID}_{i},K_{GWN-S},{ID}_{SN}$ | ${TC}_{j},{ID}_{SN}$ | 2080 |
| M.L.Das | ${ID}_{i},H\left( {PW}_{i} \right), N_{i},x_{a}$ | ${ID}_{i},K, N_{i},x_{a},S_{N}$ | $S_{N},x_{a}$ | 1472 |
| XUE et al. | ${ID}_{i},H\left( H\left( {PW}_{i} \right) \right),{TE}_{i},{PTC}_{i}$ | $K_{GWN-S},K_{GWN-U},{ID}_{SN}$ | ${TC}_{j},{ID}_{SN}$ | 1024 |
| Ours | ${PTC}_{i},V_{i},e_{i},{ID}_{GW},$ | ${PK}_{GW},{PID}_{j},{ID}_{GW},{ID}_{SC}$ | ${PTC}_{J},{PK}_{j}$ | 1328 |
