# Supplementary material for: A Temporal Credential-Based Mutual Authentication with Multiple-Password Scheme for Wireless Sensor Networks
Source: PLoS One. 2017 Jan 30;12(1):e0170657. doi: 10.1371/journal.pone.0170657 (PMC5279753; doi:10.1371/journal.pone.0170657)
Supplement: S6 Table — This table illustrates the comparison with other schemes. The detailed comparison shows that the communication overhead accounts for the majority of total overhead. (DOCX) [file pone.0170657.s006.docx]

**Table 6. Comparison of total consumption**

| schemes | Rough total consumption($\mathrm{mJ}$) | | | | | | | | | |  |
| --- | --- | --- | --- | --- | --- | --- | --- | --- | --- | --- | --- |
|  | $U$ | | | $\mathrm{GW}$ | | | $\mathrm{SN}$ | | | total | % |
|  | CC | PC | Tot | CC | PC | Tot | CC | PC | Tot |  |  |
| Nam et al. | 4.463 | 0.864 | 5.327 | 4.645 | 0.465 | 5.11 | 2.206 | 0.035 | 2.241 | 12.678 | 89.24 |
| A.K.Das | 4.7 | 0.476 | 5.176 | 9.132 | 0.084 | 9.216 | 3.643 | 0.038 | 3.681 | 18.073 | 96.69 |
| He et al. | 5.489 | 0.061 | 5.55 | 6.093 | 0.069 | 6.162 | 4.03 | 0.046 | 4.076 | 15.788 | 98.89 |
| Jiang et al. | 4.622 | 0.054 | 4.676 | 8.923 | 0.069 | 8.992 | 3.643 | 0.038 | 3.681 | 17.349 | 99.07 |
| M.L.Das | 2.299 | 0.03 | 2.329 | 3.151 | 0.03 | 3.181 | 0.852 | 0.008 | 0.86 | 6.37 | 98.93 |
| XUE et al. | 5.489 | 0.077 | 5.566 | 6.093 | 0.084 | 6.177 | 4.03 | 0.046 | 4.076 | 15.819 | 98.69 |
| Ours | 4.955 | 0.069 | 5.024 | 4.883 | 0.054 | 4.937 | 3.251 | 0.038 | 3.289 | 13.25 | 98.41 |
